# Olanzapine’s Cytogenetic Effect on T Lymphocytes in Systemic Lupus Erythematosus and Rheumatoid Arthritis Patients: In Vitro Study

**DOI:** 10.7759/cureus.37683

**Published:** 2023-04-17

**Authors:** Georgios Demirtzoglou, Sofia-Ifigeneia Chrysoglou, Theodora Katopodi, Theodoros Dimitroulas, Zafeiroula Iakovidou-Kritsi, Alexandros Garyfallos, Alexandros Lambropoulos

**Affiliations:** 1 1st Laboratory of Medical Biology and Genetics, School of Medicine, Faculty of Health Sciences (FHS) of Aristotle University of Thessaloniki, Thessaloniki, GRC; 2 2nd Department of Internal Medicine, 251 General Airforce Hospital, Athens, GRC; 3 4th Department of Internal Medicine, Hippokration General Hospital, Thessaloniki, GRC

**Keywords:** proliferation rate index, sister chromatid exchanges, cytogenetic studies, mitotic index, rheumatoid arthritis, systemic lupus erythematosus, olanzapine

## Abstract

Objectives: This study will investigate olanzapine’s cytogenetic behavior in cultured human T lymphocytes in patients with systemic lupus erythematosus (SLE) and rheumatoid arthritis (RA).

Methods: Three olanzapine solutions were added in cultures of peripheral blood lymphocytes of healthy individuals, SLE, and RA patients. After 72 hours of incubation, the cultured lymphocytes were plated on glass slides and stained with the fluorescence plus Giemsa method. Sister chromatid exchanges (SCEs), proliferation rate index (PRI), and mitotic index (MI) were measured with the optical microscope.

Results: There was a statistically significant (p=0.001) dose-dependent increase of SCEs in SLE and RA patients compared to healthy individuals and a statistically significant (p=0.001) reduction of PRI and MI in the highest concentration in the SLE group. Moreover, Spearman's rank correlation coefficient was applied to calculate the correlation between SCEs, PRI, and MI. Negative significant correlations were noticed for both patient groups concerning SCEs-PRI alterations and SCEs-MI alterations. Conversely, positive correlations were noticed for both patient groups for PRI-MI alterations.

Conclusions: Olanzapine affects T lymphocytes from SLE and RA patients by modifying DNA replication procedures and DNA damage response. Considering the use of olanzapine in neuropsychiatric symptoms of SLE, further in vivo studies are necessary to evaluate its effect on human DNA.

## Introduction

Olanzapine, an atypical antipsychotic drug, is approved for treating schizophrenia, bipolar disorder, and other psychiatric disorders. It is commonly used off-label for treating other conditions, such as eating disorders [1]. It is an antagonist of the D2 dopamine and 5-HT2A serotonin receptors and has antagonist properties at M1-muscarinic, H1-histaminic, and alpha-1 adrenergic receptors [1]. Olanzapine, like other atypical antipsychotics, displays serious adverse events in patients’ metabolic profiles like weight gain and insulin resistance [1].

Systemic lupus erythematosus (SLE) is a chronic, multisystem autoimmune disorder that typi­cally affects women between puberty and menopause. It is characterized by heterogeneity in clinical presentations and can affect many organs, including the skin, joints, the central nervous system, and the kidneys [2]. Diagnosis is primarily clinical and remains challenging because of the heterogeneity of SLE. It is considered to be a multifactorial disease. Genetic and epigenetic factors, immune dysregulation, and environmental factors like ultraviolet radiation and Epstein-Barr virus infection are involved. T lymphocytes are critically involved in the development of systemic autoimmunity and organ damage in SLE [2]. DNA damage also plays a critical role in SLE pathogenesis [3].

Rheumatoid arthritis (RA) is a relapse-remitting inflammatory, autoimmune disease that primarily affects the synovial tissue and is associated with autoantibodies targeting various molecules, including modified self-epitopes. It is characterized by the presence of rheumatoid factor (RF), an autoantibody to immunoglobulin G (IgG), and autoantibodies to citrullinated proteins (anti-citrullinated protein antibodies (ACPAs)) [4]. Genetic, epigenetic, and environmental factors such as smoking, dust inhalation, and respiratory viral infections contribute to RA pathogenesis [4]. Immunogenetic studies suggest a central role of T lymphocytes in RA pathogenesis [5]. In addition, recent studies suggest that RA is associated with deficits in telomere maintenance and overall genomic instability of T lymphocytes [6].

Olanzapine’s cytogenetic behavior has been studied in vitro in lymphocytes from healthy individuals [7,8], and in vivo in lymphocytes from schizophrenia patients [1]. Olanzapine in vitro seems to have cytotoxic but not cytostatic effects on cultured lymphocytes of healthy donors [7]. Olanzapine appears cytotoxic but not cytostatic in schizophrenic patients, but the danger of cytotoxicity seems to be more prominent with known DNA-damaging agents such as smoking [1].

The present study aims to evaluate the effect of olanzapine in T lymphocyte DNA of patients with SLE and RA by calculating three sensitive cytogenetic indices: sister chromatid exchanges (SCEs), proliferation rate index (PRI), and mitotic index (MI). The SCE index refers to the exchange of genetic material between two identical sister chromatids. SCEs are believed to result from a defective DNA replication process on a damaged template, possibly at the replication fork [9]. SCE index has been identified as one of the most sensitive indices among sensitive biomarkers of genotoxicity, together with chromosomal aberrations, comet assay, and micronuclei [9]. PRI and MI have been used as sensitive indicators for evaluating the cytostatic activity of various environmental hazards or therapeutic agents. Changes in SCE frequency, PRI, and MI represent the first signs of DNA damage [9]

## Materials and methods

Ethics statement

The Ethics Committee of Aristotle University of Thessaloniki School of Medicine, Thessaloniki, Greece, approved the study (approval number: 9.657/12-7-2017). In addition, written informed consent was obtained from all individuals.

Patients and controls

The present study enrolled 30 SLE patients (25 females, five males) and 30 RA patients (20 females, 10 males) from the Rheumatology Clinic of the 4th Department of Internal Medicine of Hippokration General Hospital, Thessaloniki, Greece. SLE patients met either the revised 1997 American College of Rheumatology (ACR) classification criteria for SLE or the 2012 Systemic Lupus International Collaborating Clinics (SLICC) classification criteria for SLE. RA patients were meeting the 2010 ACR/European Alliance of Associations for Rheumatology (EULAR) RA classification criteria. Patients did not receive olanzapine as part of their treatment. SLE patients' mean age was 28±7 years; they were all anti-nuclear antibodies (ANA) positive, and 60% were anti-double strand DNA (anti-dsDNA) positive at diagnosis. RA patients' mean age was 47±6 years; they were either RF-positive or ACPA-positive. SLE patients' mean disease duration was 6±3 years, and RA patients' was 10±4 years. All patients had achieved disease remission or low to moderate disease activity. Disease activity was measured with Systemic Lupus Erythematosus Disease Activity Index (SLEDAI) score for SLE patients and Disease Activity Scores 28 (DAS28) for RA patients. All patients were treated with conventional synthetic Disease-Modifying Anti-Rheumatic Drugs (DMARDs) with or without a low dose of prednisone, and 60% of RA patients were treated with a tumor necrosis factor-alpha (TNF-a) inhibitor.

The control group was composed of 30 healthy, young, nonsmoker males (n=15) and females (n=15) (mean age 20±2 years). All volunteers in the control group did not receive any pharmaceutical substance and did not mention the new onset of symptoms of any disease for the last 15 days. Venous blood (5-7ml) collected from all study subsets was used immediately for lymphocyte culture preparation and the SCE, PRI, and MI indices calculation.

In vitro cytogenetic assays

Human lymphocyte cultures [9] were prepared by adding in 5 ml chromosome medium (RPMI-1640 (Biochrom Ltd, Cambridge, United Kingdom) supplemented with 20% fetal calf serum, 0.63% L-glutamine, 0.63% penicillin/streptomycin, and 2% phytohaemagglutinin) at the beginning of the culture life, 11-12 drops of heparinized whole peripheral blood,5-bromodeoxyuridine (BrdU) solution (5 μg/ml/culture), and olanzapine solution (A=10μg/ml or B=20μg/ml or C=40μg/ml), where A, B, and C are the first, second, and third olanzapine solutions.

T lymphocyte cultures were incubated at 37^o^C for 72 hours in a dark incubator to minimize photolysis of BrdU. Colchicine was added two hours before the end of the incubation. T lymphocytes were then collected by centrifugation and exposed to 0.075M potassium chloride (KCl) solution for 12 minutes. The hypotonic solution spreads chromosomes and causes hemolysis of red blood cells. Methanol:acetic acid (3:1) solution was used for fixing the pellet three times. Drops of a concentrated suspension of cells were placed on microslides and allowed to air dry. For SCE, PRI, and MI analysis, the slides were stained by a modification of the fluorescence plus Giemsa procedure to obtain harlequin chromosomes [9]

Statistical analysis

For SCE estimation, 30 suitably spread second-division nuclei from each culture were blindly scored. For PRI calculation, 100 nuclei were blindly scored in each culture's first, second, third, or higher divisions. PRI =M1+2M2+3M3/100, where M1, M2, and M3+ are the percent values of nuclei in the first, second, third, or higher divisions, respectively. All cell divisions in an optical field of 1000 nuclei were scored to calculate the MI index-MI=number of cells in mitosis/total number of nuclei (1000). The statistical analysis was done using IBM SPSS Statistics for Windows, Version 22.0 (Released 2013; IBM Corp., Armonk, New York, United States). All values were expressed as mean ± standard error of the mean (SEM). The Mann-Whitney U-test was used to compare values between the different groups and subgroups. The Kruskal-Wallis test was performed to evaluate the dosage effect of olanzapine on SCEs, PRI, and MI. Spearman's rank correlation coefficient was used to estimate the correlation between all cytogenetic indices in both control and patient groups.

## Results

**Figure 1:**
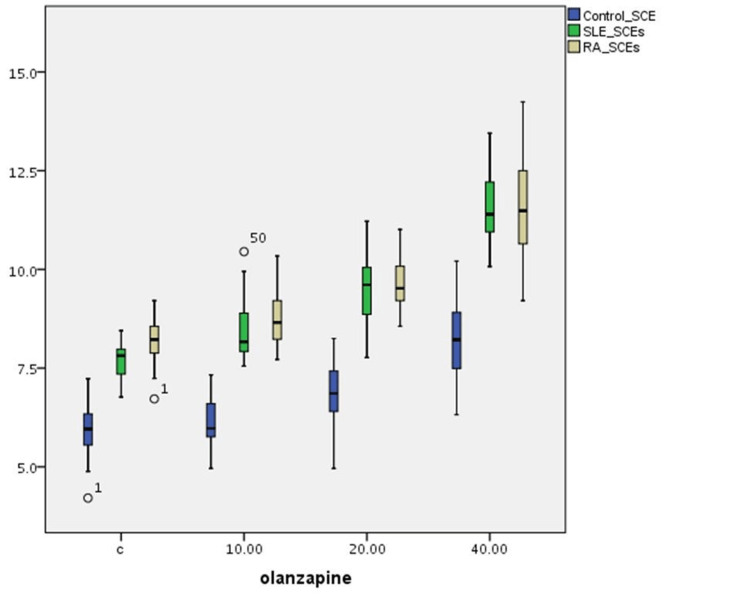
Olanzapine’s effect on SCE index variations in human T-lymphocyte cultures from healthy individuals, SLE, and RA patients SCE: sister chromatid exchange; SLE: systemic lupus erythematosus; RA: rheumatoid arthritis

The effect of the three olanzapine solution concentrations on SCE alterations in T-cultured lymphocytes of all three groups is outlined in Table 1 and Figure 1. There is a statistically significant difference in SCE frequencies between (a) the control and the SLE group (p=0.001), (b) the control and the RA group (p=0.001), and (c) the SLE and RA group (p=0.001) without the effect of olanzapine. The cultured lymphocyte SCE frequencies of patients' groups are statistically significantly increased (p=0.001) compared to those of the control group for all olanzapine concentrations. A statistically significant induction in SCE frequency is observed after the effect of 40 μg/ml olanzapine solution on the control group cultured lymphocytes (p=0.001). Furthermore, a dose-dependent induction in SCE frequency is observed in both SLE and RA groups (p=0.001).

**Table 1:** Effect of olanzapine on SCE frequency (SCE/cell±SE) in T-cultured lymphocytes of SLE, RA patients, and healthy donors p^1^ represents the p-value for the Kruskal-Wallis test, which was performed to evaluate the dosage effect of olanzapine on SCE for all groups, p^2^ represents the p-value for the Mann-Whitney U test for the comparison of the SCE index between control and SLE group for various olanzapine concentrations, p^3^ represents the p-value for the Mann-Whitney U test for the comparison of the SCE index between control and RA group for various olanzapine concentrations, p^4^ represents the p-value for the Mann-Whitney U test for the comparison of the SCE index between RA and SLE group for various olanzapine concentrations. SCE: sister chromatid exchange; SE: standard error; SLE: systemic lupus erythematosus; RA: rheumatoid arthritis

Olanzapine (μg/ml)	0	10	20	40	p^1^
Healthy (n=30)	5.97±0.12	6.12±0.11	6.86±0.14	8.27±0.20	0.001
SLE (n=30)	7.69±0.08	8.43±0.13	9.51±0.17	11.57±0.16	0.001
RA (n=30)	8.19±0.27	8.79±0.28	9.65±0.27	11.58±0.31	0.001
p^2^	0.001	0.001	0.001	0.001	
p^3^	0.001	0.001	0.001	0.001	
p^4^	0.001	0.038	0.679	0.871	

**Figure 2:**
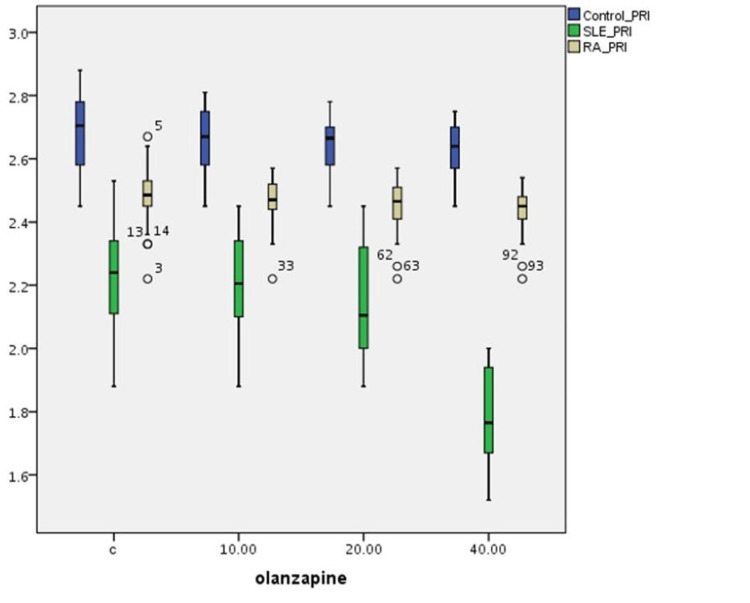
Olanzapine’s effect on PRI index variations in human T-lymphocyte cultures from healthy individuals, SLE, and RA patients PRI: proliferation rate index;  SLE: systemic lupus erythematosus; RA: rheumatoid arthritis

The effect of the three olanzapine solution concentrations on PRI alterations in T-cultured lymphocytes of all three groups is outlined in Table 2 and Figure 2. There is a statistically significant difference in PRI frequencies between (a) the control and the SLE group (p=0.001), (b) the control and the RA group (p=0.001), and (c) the SLE and RA group (p=0.001) without olanzapine. PRI differences between these groups are statistically significant (p=0.001) for various olanzapine concentrations too. A statistically significant reduction in PRI frequency is observed in 40 μg/ml olanzapine concentration for the SLE group (p=0.001). No significant difference in PRI variations is observed among different concentrations of olanzapine in the control and RA groups.

**Table 2:** Effect of olanzapine on PRI frequency (PRI±SE) in T lymphocyte cultures of SLE, RA patients, and healthy donors p^1^ represents the p-value for the Kruskal-Wallis test, which was performed to evaluate the dosage effect of olanzapine on the PRI index for all groups, p^2^ represents the p-value for the Mann-Whitney U test for the comparison of the PRI index between control and SLE group for various olanzapine concentrations, p^3^ represents the p-value for the Mann-Whitney U test for the comparison of the PRI index between control and RA group for various olanzapine concentrations, p^4^ represents the p-value for the Mann-Whitney U test for the comparison of the PRI index between RA and SLE group for various olanzapine concentrations. PRI: proliferation rate index; SCE: sister chromatid exchange; SE: standard error; SLE: systemic lupus erythematosus; RA: rheumatoid arthritis

Olanzapine (μg/ml)	0	10	20	40	p^1^
Healthy (n=30)	2.69±0.020	2.65±0.018	2.62±0.015	2.59±0.015	0.073
SLE (n=30)	2.23±0.028	2.20±0.030	2.15±0.031	1.80±0.026	0.001
RA (n=30)	2.48±0.016	2.44±0.015	2.45±0.016	2.43±0.014	0.127
p^2^	0.001	0.001	0.001	0.001	
p^3^	0.001	0.001	0.001	0.001	
p^4^	0.001	0.001	0.001	0.001	

**Figure 3:**
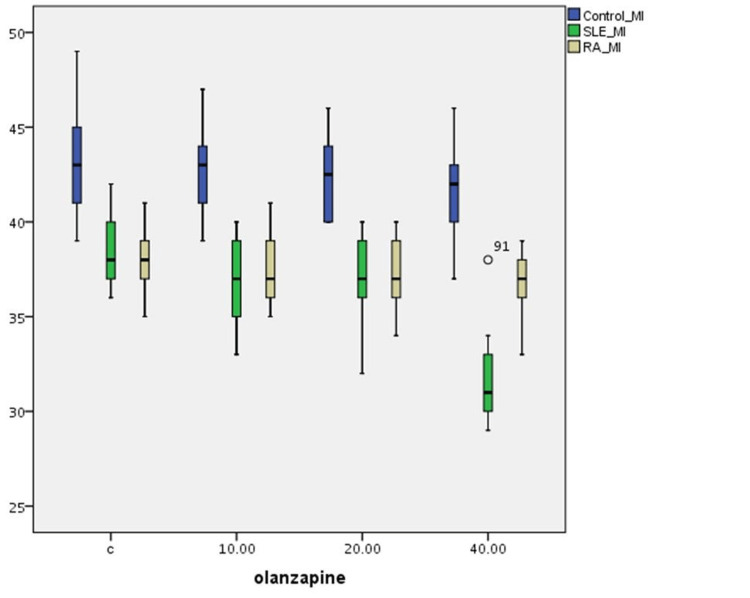
Olanzapine’s effect on MI index variations in human T-lymphocyte cultures from healthy individuals, SLE, and RA patients MI: mitotic index; SLE: systemic lupus erythematosus; RA: rheumatoid arthritis

The effect of the three olanzapine solution concentrations on SCE alterations in T-cultured lymphocytes of all three groups is outlined in Table 3 and Figure 3. A statistically significant difference is observed in MI frequencies between (a) the control and the SLE group (p=0.001) and (b) the control and the RA group (p=0.001) without olanzapine, whereas the difference between the SLE and the RA group is not statistically significant (p=0.086). MI differences between the control and patient groups are statistically significant (p=0.001) for various olanzapine concentrations. However, MI differences between SLE and RA groups are not statistically significant for olanzapine concentrations of 10 μg/ml and 20 μg/ml. For the highest olanzapine concentration, MI values are different at a statistically significant level. Moreover, a statistically significant reduction in MI frequency is observed in 40 μg/ml olanzapine concentration for the SLE group (p=0.001). No significant difference in MI variations is observed among different concentrations of olanzapine in the control and RA groups.

**Table 3:** Effect of olanzapine on MI frequency (MI±SE) in T lymphocyte cultures of SLE, RA patients, and healthy donors p^1^ represents the p-value for the Kruskal-Wallis test, which was performed to evaluate the dosage effect of olanzapine on the MI index for all groups, p^2^ represents the p-value for the Mann-Whitney U test for the comparison of the MI index between control and SLE group for various olanzapine concentrations, p^3^ represents the p-value for the Mann-Whitney U test for the comparison of the MI index between control and RA group for various olanzapine concentrations, p^4^ represents the p-value for the Mann-Whitney U test for the comparison of the MI index between RA and SLE group for various olanzapine concentrations. MI: mitotic index; SE: standard error; SLE: systemic lupus erythematosus; RA: rheumatoid arthritis

Olanzapine (μg/ml)	0	10	20	40	p^1^
Healthy (n=30)	42.90±0.44	42.73±0.37	42.30±0.35	41.67±0.41	0.187
SLE (n=30)	38.53±0.31	36.77±0.39	37.17±0.37	31.63±0.40	0.001
RA (n=30)	37.73±0.27	37.47±0.28	37.17±0.27	36.57±0.31	0.078
p^2^	0.001	0.001	0.001	0.001	
p^3^	0.001	0.001	0.001	0.001	
p^4^	0.086	0.301	0.863	0.001	

Table 4 illustrates the values and statistical significance of correlation rates between SCE, PRI, and MI. For the control group, no statistically significant correlations are observed. For the SLE group, a statistically significant negative correlation (p=0.001) is observed between SCE and PRI (-0.580) and between SCE and MI (-0.615). On the other hand, a statistically significant (p=0.001) positive correlation is also observed between PRI and MI (0.611). For the RA group, a statistically significant negative correlation (p<0.05) is observed between SCE and PRI (-0.231) and between SCE and MI (-0.300). On the other hand, a statistically significant (p=0.01) positive correlation between PRI and MI (0.312) is also observed.

**Table 4:** Values and statistical significance of correlation rates between SCE, PRI, and MI 1Spearman's rho SCE: sister chromatid exchange; PRI: proliferation rate index; MI: mitotic index; SLE: systemic lupus erythematosus; RA: rheumatoid arthritis

Correlation	Value	p^1^
SCE–PRI (Healthy)	-0.066	0.472
SCE–MI (Healthy)	-0.083	0.365
PRI–ΜΙ (Healthy)	0.034	0.714
SCE–PRI (SLE)	-0.580	0.001
SCE–ΜΙ (SLE)	-0.615	0.001
PRI–ΜΙ (SLE)	0.611	0.001
SCE–PRI (RA)	-0.231	0.011
SCE–ΜΙ (RA)	-0.300	0.001
PRI–ΜΙ (RA)	0.312	0.01

## Discussion

Olanzapine's cytotoxicity in healthy individuals at high doses in vitro has been studied [7], but there is no evidence concerning its cytogenetic behavior in T lymphocytes from SLE and RA patients. In the present study, an interesting cytogenetic behavior of olanzapine is observed. Olanzapine in vitro seems cytotoxic in the healthy group at the concentration of 40 μg/ml. In contrast, it shows cytotoxic activity in patient groups at lower doses, equal to the therapeutic doses used for treating psychosis. In addition, T lymphocytes from SLE patients seem to be affected at lower olanzapine doses compared to T lymphocytes from RA patients (Figure 1).

Furthermore, though olanzapine is not cytostatic in healthy individuals and RA patients, it seems to be cytostatic at high concentrations in SLE patients (Figures 2, 3). Differences in the three cytogenetic indices are observed in patient groups before the effect of olanzapine compared to those of the control group. This is probably related to DNA damage implicated in the pathogenesis of the two diseases, the effect of patients' standard treatment, and the increased age of RA patients.

DNA damage response mechanisms and DNA damage repair potentially play a central role in SLE [3] and RA [10] pathogenesis. Loss of tolerance to self-DNA and anti-DNA antibody formation is the hallmark of SLE pathogenesis. Moreover, SLE patients have difficulty maintaining genome stability and DNA repair mechanisms [3] T lymphocytes of SLE patients seem to be affected not only by defects of DNA repair mechanisms but also by excessive DNA damage, which could be the reasons that cultured lymphocytes from SLE patients have higher SCE frequencies compared to healthy individuals [11,12] as it is confirmed in the present study. Oxidative stress and superoxide dismutase decreased activity could induce direct DNA damage in SLE patients [3]. Nucleotide excision repair and DNA double-strand breaks repair mechanisms are defective and related to apoptosis in SLE patients [3]. Downregulation of genes encoding proteins involved in the nucleoxide excision repair pathway in SLE patients has been found. Such proteins are tDNA damage-binding protein 1 (DDB1), ERCC2, Xeroderma pigmentosum complementation group A (XPA), and xeroderma pigmentosum complementation group C (XPC). Many proteins that relate to the DNA repair mechanism are targets of autoantibodies. For example, two subunits of the Ku protein, the poly(ADP-ribose) polymerase 1 (PARP-1) protein, the catalytic subunit of the DNA-dependent protein kinase (DNA-PK) and the DNA ligase IV are targets for autoantibodies and related to DNA double-strand breaks repair deficiency [3]. These mechanisms seem defective in active disease and remission states, but active SLE may correlate with more defective DNA repair [3,13]. Souliotis et al. also observed a strong correlation between the accumulation of DNA damage and increased apoptosis rates in SLE patients' white blood cells [3].

The present research also shows a statistically significant correlation between DNA alterations and cytostaticity (Table 4). Studies show nucleotide excision repair could be related to defective chromatin organization in SLE patients [3]. SLE is characterized by more condensed local chromatin, which stops DNA repair enzymes from accessing DNA-damaged sites [10]. DNA histone acetylation modifications could be involved in defective DNA repair, but results from studies examining histone modifications in SLE patients have been controversial [3]. A global hypoacetylation of histone H3 and H4, a downregulation in mRNA of histone acetyltransferases (P300, CREBBP), and an increase of sirtuin 1 (histone deacetylase) mRNA level was observed in active SLE CD4+ T lymphocytes in vitro compared with controls [14]. This finding indicates that epigenetic modifications could be central to SLE DNA damage repair.

Previous studies show that SCEs in human cultured T lymphocytes from patients with RA do not seem to be elevated compared to SLE patients and controls [11,14]. In the present study, the differences in SCE frequency of RA patients compared to healthy individuals could reflect the effect of standard treatment, increased age, and disease activity [11,15]. Methotrexate, the most commonly used DMARD in RA treatment, is a folic acid antagonist. However, studies have shown its genotoxic behavior (SCE and chromosomal aberrations induction) in cultured lymphocytes from healthy individuals via induction of oxidative stress [16]. In addition, cytokine production and intracellular signal transduction depend on disease activity and can alter the SCE index and proliferation capacity [15].

However, DNA damage also seems essential in RA pathogenesis [10,17]. Recent studies indicate that age is an essential endogenous factor that affects the level of genomic damage and the capacity of cells to replicate. A correlation between increased SCEs and age was found [18]. Aging of T lymphocytes (telomere fragility and attrition) and DNA damage repair system insufficiency may promote inflammation in RA [17]. T lymphocytes are sensitive to age-related abnormalities because of the intense replication pressure they are exposed to [1]. T lymphocytes from RA patients seem to malfunction in three interconnected domains related to a premature aging process [17]. The three malfunctioned domains are DNA damage repair, metabolic activity-energy generation, and plasma membrane shaping. DNA damage response is a hierarchical network that includes cell cycle checkpoints, DNA repair, and DNA-damage tolerance pathways, and it closely relates to premature aging in RA [6]. Recent studies investigating DNA damage response network in the pathogenesis of RA showed oxidative stress, prolonged stalling in DNA replication fork progression that indicates replication stress, chromosome misaggregation events, telomere shortening, and chromothripsis that leads to micronuclei formation [10]. Global genome repair, a subpathway of nucleotide excision repair (one of the six major DNA repair pathways), seems insufficient in RA patients, probably due to the more condensed chromatin structure than normal controls [13]. Despite this evidence, the mechanism of inefficient DNA repair in RA patients still needs to be thoroughly understood, and the role of genetic and epigenetic factors is still being determined.

Although olanzapine's mechanism of action is poorly understood, it is possibly related to immune system regulation. Studies show that many patients diagnosed with schizophrenia exhibit low-grade central and peripheral inflammation [19]. Dysregulation of the inflammatory response includes genetic and environmental factors such as toxins, viruses, and bacteria [19]. Inflammation in the central nervous system is probably mediated by pro-inflammatory cytokines, microglia, astrocytes, and T and B lymphocytes [19]. Studies also found that the levels of pro-inflammatory cytokines are increased in the blood and cerebrospinal fluid of patients with schizophrenia [19].

Moreover, schizophrenia susceptibility and treatment response to olanzapine are associated with two human leukocyte antigen (HLA) locus single nucleotide polymorphisms (SNPs) [20]. Better treatment response to olanzapine is related to a double amino acid variant at positions 62 and 66 of the peptide binding groove of the HLA-A molecule [20]. This observation indicates that olanzapine is probably central to immune dysregulation treatment in schizophrenia. Furthermore, olanzapine seems to have marginal immunomodulatory effects in drug naïve patients in the first episode of psychosis, regulating the expression of genes related to the immune system, such as IL-17A and IL-20 [21]. In vivo, cytogenetic studies showed that olanzapine affects T lymphocytes, and long-term olanzapine use and smoking may increase SCE frequency [1].

Olanzapine's cytotoxicity may be related to its chemical structure. Recent studies indicate that drugs like olanzapine, which have a piperazine ring, can be cytotoxic for human lymphocytes via oxidative stress induction and mitochondrial and lysosomal damage [22]. Olanzapine induced oxidative stress, organelle damage, chromatin disorganization, and DNA damage response alterations. Therefore, it may explain its cytotoxic in lower concentrations in patient groups compared to the control group. Furthermore, olanzapine may modulate epigenetic mechanisms related to DNA damage response and DNA repair system [23,24]. DNA methylation signature [24] and histone acetylation [23] may be altered by olanzapine. Olanzapine seems to induce histone deacetylases (HDACs) expression in animal models in vivo in different brain parts. HDAC1-3, HDAC5, and HDAC8 seem to be overexpressed due to olanzapine's effect. Yang et al. showed that one of the most important drugs in SLE treatment, mycophenolic acid, can reduce HDAC2 levels. In contrast, olanzapine can induce HDAC2 expression [25]. These epigenetic mechanisms play a central role in SLE and RA pathogenesis, which may explain the differences between patient and control groups. The common mechanisms by which olanzapine may affect SLE and RA lymphocytes align with the results of the present study, as no statistically significant difference was found between SLE and Ra groups concerning SCE.

Inhibition of extensive autophagy may be related to high-dose olanzapine's cytostatic behavior in cultured T lymphocytes from SLE patients. Extensive autophagy can contribute to apoptosis or function as an alternative cell death pathway [26]. Evidence shows that olanzapine in vivo can stimulate extensive autophagy via an mTOR-independent pathway, which involves Forkhead box class O family member proteins (FoxO) transcription factors and oxidative stress [26]. Different members of FoxO and their phosphorylation status play a central role in immune cell homeostasis and survival in SLE [27]. Moreover, olanzapine seems to stimulate the activation of various protein kinase signaling pathways, including Akt/protein kinase B (PKB), extracellular-regulated kinase (ERK), ERK1/2, and mitogen-activated protein kinase (MAPK), p38 in PC12 cells [28]. Correlation between SCE induction and PRI and MI reduction possibly indicates a common pathogenic mechanism and oxidative stress induced by olanzapine may be the first step in T lymphocyte damage in SLE.

Interestingly, Türkez and Toğar did not prove a cytotoxic effect of olanzapine in cultured lymphocytes from healthy individuals in vitro, probably due to erythrocytes in the culture medium [8]. However, erythrocytes could have acted like antioxidants in their in vitro model because they contain glutathione, glutathione peroxidase, and glutathione-S-transferase [29]. This probably indicates the central role of SCE induction by the effect of olanzapine. Furthermore, olanzapine at low doses and for a short treatment period showed radioprotective and anti-apoptotic effects in normal human lymphocytes in vitro, acting as a potent antioxidant agent [30]. This observation points to the necessity of further in-depth investigation of the cytogenetic behavior of olanzapine.

Limitations of the study

The study's limitations have to be considered to understand its results. Sample heterogeneity concerning population age and sex, lack of correlation with clinical indices and treatment, acute dosing of olanzapine, and in vitro study conduction are the most important limitations. Furthermore, subtherapeutic doses of olanzapine were not tested, molecular cytogenetic assays were not conducted, and only a single lymphocyte population was tested.

## Conclusions

Olanzapine seems cytotoxic in high concentrations in SLE and RA patients and control groups, but lymphocytes from both patient groups are affected in lower concentrations. No cytostatic properties for lymphocytes from healthy individuals and RA patients were noticed, but in high concentrations, olanzapine has cytostatic effects on cultured lymphocytes from SLE patients in vitro. Oxidative stress and DNA damage control system play an essential role. This cytogenetic behavior needs further in vitro and in vivo investigation in other cell lines combined with molecular cytogenetic techniques.
